# Supplementary material for: Baseline dynamics of Symbiodiniaceae genera and photochemical efficiency in corals from reefs with different thermal histories
Source: PeerJ. 2023 Jun 1;11:e15421. doi: 10.7717/peerj.15421 (PMC10239617; doi:10.7717/peerj.15421)
Supplement: Supplemental Information 1 [file peerj-11-15421-s001.docx]

**Supporting information**

**Baseline dynamics of Symbiodiniaceae genera and photochemical efficiency in corals from reefs with different thermal histories**

Crystal J. McRae^1^, Shashank Keshavmurthy^2^, Hung-Kai Chen^1^, Zong-Min Ye^1^, Pei-Jie Meng^1,3^, Sabrina L. Rosset^4^, Wen-Bin Huang^5^, Chaolun Allen Chen^2^, Tung-Yung Fan^1,6^, Isabelle M. Côté^7^

^1^ National Museum of Marine Biology and Aquarium, Pingtung, Taiwan.

^2^ Biodiversity Research Center, Academia Sinica, Taipei, Taiwan.

^3^ Graduate Institute of Marine Biology, National Dong Hwa University, Pingtung, Taiwan.

^4^ School of Biological Sciences, Victoria University of Wellington, Wellington, New Zealand

^5^ Department of Natural Resources and Environmental Studies, National Dong Hwa University, Hualien, Taiwan.

^6^ Department of Marine Biotechnology and Resources, National Sun Yat-sen University, Kaohsiung, Taiwan

^7^ Department of Biological Sciences, Simon Fraser University, British Columbia, Canada.

Corresponding authors:

Tung-Yung Fan^1,6^; Crystal J. McRae

Email address: tyfan@nmmba.gov.tw; crystal.j.mcrae@gmail.com

**
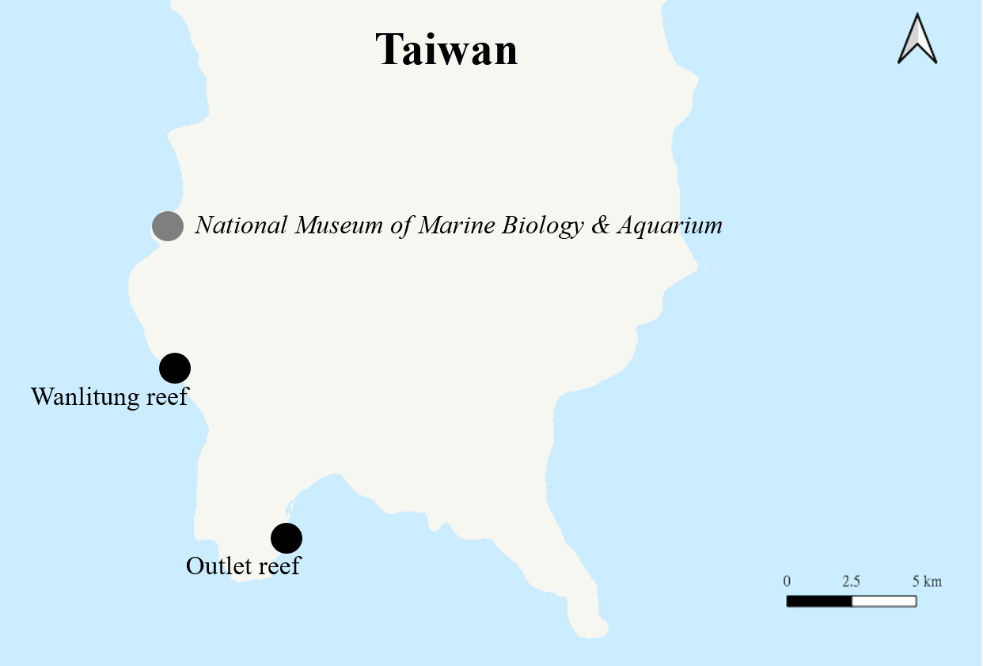
**

**Figure S1.** Map of coral collection sites, Wanlitung reef (thermally stable site; 21.955°E, 120.766°N) and Outlet reef (chronically warmed and thermally variable site; 21.931°E, 120745°N), and the research facilities of the National Museum of Marine Biology & Aquarium in southern Taiwan.

**Table S1**. Summary of individual colony sampling and condition across seasons for Outlet reef from July 2018 to August 2019. Check mark = fragments were sampled from the colony; NA = not sampled; PM = partial mortality; PB = partial bleaching; R = recovery; M = mortality. Mortality and bleaching percentages are reported relative to initial colony condition; recovery percentages are reported relative to previous sampling time point.

| Colony ID | Summer  2018 | Fall  2018 | Winter  2019 | Spring  2019 | Summer  2019 | Included in  qPCR analysis | Comments |
| --- | --- | --- | --- | --- | --- | --- | --- |
| *Pocillopora acuta* |  |  |  |  |  |  |  |
| PA1 | ✓ | ✓ | ✓ | ✓ | ✓ | ✓ |  |
| PA2 | ✓ | ✓ | ✓ | ✓ | ✓ | ✓ |  |
| PA3 | ✓ | ✓ | ✓ PM (~20%) | ✓ PM (~40%) | ✓ R (Spring + ~20%) | 🗶 |  |
| PA4 | ✓ | ✓ | ✓ | ✓ | ✓ | 🗶 |  |
| PA5 | ✓ | ✓ | ✓ | ✓ | ✓ | ✓ |  |
| PA6 | ✓ | ✓ | ✓ | ✓ | ✓ | 🗶 |  |
| PA7 | ✓ | ✓ | ✓ | ✓ | ✓ | ✓ |  |
| PA8 | ✓ | ✓ PM (~30%) | ✓ PM (~30%) | ✓ PM (~30%) | ✓ R (Spring + ~10%) | 🗶 |  |
| PA9 | ✓ | ✓ | ✓ | ✓ | ✓ | ✓ |  |
| PA10 | ✓ | ✓ | ✓ | ✓ | ✓ | ✓ |  |
| *Acropora nana* |  |  |  |  |  |  |  |
| AN1 | ✓ | NA | NA | NA | NA | NA | not relocated |
| AN2 | ✓ | NA | NA | NA | NA | NA | not relocated |
| AN3 | NA | NA | NA | NA | NA | NA | species misidentified |
| AN4 | ✓ | ✓ | ✓ | ✓ | ✓ | 🗶 |  |
| AN5 | ✓ | ✓ PB (~20%) | ✓ PM (~60%) | ✓ R (Winter + ~5%) | ✓ R (Spring +~10%) | ✓ |  |
| AN6 | NA | NA | NA | NA | NA | NA | species misidentified |
| AN7 | NA | NA | NA | NA | NA | NA | species misidentified |
| AN8 | ✓ | ✓ | ✓ | ✓ | ✓ | ✓ |  |
| AN9 | ✓ | ✓ | ✓ | ✓ | ✓ | ✓ |  |
| AN10 | ✓ | ✓ | ✓ | ✓ | ✓ | ✓ |  |
| AN11 | ✓ | ✓ | ✓ | ✓ | ✓ | 🗶 |  |
| AN12 | ✓ | ✓ | ✓ | ✓ | ✓ | ✓ |  |
| AN13 | ✓ | ✓ | ✓ | ✓ | ✓ | ✓ |  |
| AN14 | NA | NA | ✓ | ✓ | ✓ | 🗶 |  |
| AN15 | NA | NA | NA | ✓ | ✓ | 🗶 |  |
| AN16 | NA | NA | NA | ✓ | ✓ | 🗶 |  |
| *Porites lutea* |  |  |  |  |  |  |  |
| PL1 | ✓ | ✓ | ✓ | ✓ | ✓ | ✓ | < 5 m from PL2 |
| PL2 | ✓ | ✓ | ✓ | ✓ | ✓ | 🗶 | < 5 m from PL1 |
| PL3 | ✓ | ✓ | ✓ | ✓ | ✓ | 🗶 |  |
| PL4 | ✓ | ✓ | ✓ | ✓ | ✓ | ✓ |  |
| PL5 | ✓ | ✓ | ✓ | ✓ | ✓ | ✓ |  |
| PL6 | ✓ | ✓ | ✓ | ✓ | ✓ | ✓ | < 5 m from PL7 |
| PL7 | ✓ | ✓ | ✓ | ✓ | ✓ | 🗶 | < 5 m from PL6 |
| PL8 | ✓ | ✓ | ✓ | ✓ | ✓ | ✓ |  |
| PL9 | ✓ | ✓ | ✓ | ✓ | ✓ | ✓ |  |
| PL10 | ✓ | ✓ | ✓ | ✓ | ✓ | 🗶 |  |
| PL11 | NA | ✓ | ✓ | ✓ | ✓ | 🗶 |  |

**Table S2**. Summary of individual colony sampling and condition across seasons for Wanlitung reef from July 2018 to August 2019. Check mark = fragments were sampled from the colony; NA = not sampled; PM = partial mortality; PB = partial bleaching; R = recovery; M = mortality. Mortality and bleaching percentages are reported relative to initial colony condition; recovery percentages are reported relative to previous sampling time point.

| Colony ID | Summer  2018 | Fall  2018 | Winter  2019 | Spring  2019 | Summer  2019 | Included in  qPCR analysis | Comments |
| --- | --- | --- | --- | --- | --- | --- | --- |
| *Pocillopora acuta* |  |  |  |  |  |  |  |
| PA1 | ✓ | ✓ PM (~90%) | M | NA | NA | ✓ |  |
| PA2 | ✓ | M | NA | NA | NA | NA |  |
| PA3 | ✓ | ✓ | ✓ | ✓ | ✓ | ✓ |  |
| PA4 | NA | NA | NA | NA | NA | NA | species misidentified |
| PA5 | ✓ | NA | NA | NA | NA | ✓ | not relocated |
| PA6 | ✓ | NA | NA | NA | NA | NA | not relocated |
| PA7 | ✓ | ✓ | ✓ | ✓ | ✓ | ✓ |  |
| PA8 | ✓ | NA | ✓ | ✓ PB (~10%) | ✓ | 🗶 | not relocated in fall |
| PA9 | ✓ | ✓ PM (~75%) | ✓ PM (~90%) | M | NA | ✓ |  |
| PA10 | ✓ | ✓ PM (~50%) | ✓ PM (~80%) | ✓ PM (~85%) | ✓ R (Spring + ~5%) | ✓ |  |
| PA11 | NA | ✓ PB (~5%) | ✓ PM (~20%) | ✓ PM (~20%) | ✓ R (Spring + ~15%) | ✓ |  |
| PA12 | NA | ✓ | ✓ | ✓ | ✓ R (Spring + ~10%) | 🗶 |  |
| PA13 | NA | ✓ | ✓ | ✓ PM (~30%) | ✓ PM (~30%) | ✓ |  |
| PA14 | NA | ✓ PB (~10%) | ✓ PM (~30%) | ✓ PM (~60%) | M | 🗶 |  |
| PA15 | NA | ✓ | ✓ PM (~20%) | ✓ PM (~30%) | ✓ PM (~80%) | ✓ |  |
| PA16 | NA | NA | ✓ | ✓ PM (~10%) | ✓ PM (~10%) | ✓ |  |
| *Acropora nana* |  |  |  |  |  |  |  |
| AN1 | ✓ | ✓ | ✓ PM (50%) | ✓ PM (75%) | ✓ PM (75%) | ✓ |  |
| AN2 | ✓ | ✓ PM (~90%) | ✓ | ✓ | ✓ | 🗶 | < 5 m from AN8 |
| AN3 | ✓ | ✓ | ✓ | ✓ | ✓ | ✓ | < 5 m from AN13 |
| AN4 | NA | NA | NA | NA | NA | NA | species misidentified |
| AN5 | ✓ | ✓ | ✓ | ✓ | NA | ✓ | not relocated in summer 2019 |
| AN6 | ✓ | ✓ | ✓ | ✓ | ✓ | ✓ | < 5 m from AN11 & 14 |
| AN7 | ✓ | ✓ | ✓ PM (80%) | ✓ PM (80%) | M | ✓ | < 5 m from AN14 |
| AN8 | ✓ | ✓ | ✓ | ✓ | ✓ | ✓ |  |
| AN9 | ✓ | ✓ PM (~40%) | ✓ PM (40%) | ✓ PM (50%) | ✓ PM (50%) | 🗶 |  |
| AN10 | ✓ | ✓ | ✓ PM (40%) | ✓ PM (60%) | M | ✓ |  |
| AN11 | NA | ✓ | ✓ | ✓ | ✓ | 🗶 | < 5 m from AN6 & 14 |
| AN12 | NA | NA | NA | NA | NA | NA | species misidentified |
| AN13 | NA | ✓ | ✓ | ✓ | ✓ | 🗶 | < 5 m from AN3 |
| AN14 | NA | ✓ | ✓ | ✓ | ✓ | ✓ | < 5 m from AN7 |
| AN15 | NA | ✓ | ✓ | ✓ | ✓ | 🗶 | < 5 m from AN6 & 11 |
| *Porites lutea* |  |  |  |  |  |  |  |
| PL1 | ✓ | ✓ | ✓ | ✓ | ✓ | ✓ |  |
| PL2 | ✓ | ✓ | ✓ | ✓ | ✓ | ✓ |  |
| PL3 | ✓ | ✓ | ✓ | ✓ | ✓ | ✓ |  |
| PL4 | ✓ | ✓ | ✓ | ✓ | ✓ | ✓ |  |
| PL5 | ✓ PM (~35%) | ✓ PM (~35%) | ✓ PM (~35%) | ✓ PM (~35%) | ✓ PM (~35%) | 🗶 |  |
| PL6 | ✓ PM (~5%) | ✓ PM (~5%) | ✓ PM (~5%) | ✓ PM (~5%) | ✓ PM (~5%) | ✓ |  |
| PL7 | ✓ | ✓ | ✓ | ✓ | ✓ | 🗶 |  |
| PL8 | ✓ PM (~20%) | ✓ PM (~20%) | ✓ PM (~20%) | ✓ PM (~20%) | ✓ PM (~20%) | 🗶 |  |
| PL9 | ✓ PM (~60%) | ✓ PM (~70%) | ✓ PM (~80%) | ✓ PM (~90%) | ✓ R (Spring + ~10%) | 🗶 |  |
| PL10 | ✓ | ✓ | ✓ | ✓ | ✓ | ✓ |  |

**Table S3**. Summary of ‘qPCR analysis subset’ colony condition across seasons for Outlet reef from July 2018 to August 2019. Check mark = fragments were sampled from the colony; NA = not sampled; PM = partial mortality; PB = partial bleaching; R = recovery; M = mortality. Mortality and bleaching percentages are reported relative to initial colony condition; recovery percentages are reported relative to previous sampling time point.

| Colony ID | Summer  2018 | Fall  2018 | Winter  2019 | Spring  2019 | Summer  2019 |
| --- | --- | --- | --- | --- | --- |
| *Pocillopora acuta* |  |  |  |  |  |
| PA1 | ✓ | ✓ | ✓ | ✓ | ✓ |
| PA2 | ✓ | ✓ | ✓ | ✓ | ✓ |
| PA5 | ✓ | ✓ | ✓ | ✓ | ✓ |
| PA7 | ✓ | ✓ | ✓ | ✓ | ✓ |
| PA9 | ✓ | ✓ | ✓ | ✓ | ✓ |
| PA10 | ✓ | ✓ | ✓ | ✓ | ✓ |
| *Acropora nana* |  |  |  |  |  |
| AN5 | ✓ | ✓ PB (~20%) | ✓ PM (~60%) | ✓ R (Winter + ~5%) | ✓ R (Spring +~10%) |
| AN8 | ✓ | ✓ | ✓ | ✓ | ✓ |
| AN9 | ✓ | ✓ | ✓ | ✓ | ✓ |
| AN10 | ✓ | ✓ | ✓ | ✓ | ✓ |
| AN12 | ✓ | ✓ | ✓ | ✓ | ✓ |
| AN13 | ✓ | ✓ | ✓ | ✓ | ✓ |
| *Porites lutea* |  |  |  |  |  |
| PL1 | ✓ | ✓ | ✓ | ✓ | ✓ |
| PL4 | ✓ | ✓ | ✓ | ✓ | ✓ |
| PL5 | ✓ | ✓ | ✓ | ✓ | ✓ |
| PL6 | ✓ | ✓ | ✓ | ✓ | ✓ |
| PL8 | ✓ | ✓ | ✓ | ✓ | ✓ |
| PL9 | ✓ | ✓ | ✓ | ✓ | ✓ |

**Table S4**. Summary of ‘qPCR analysis subset’ colony condition across seasons for Wanlitung reef from July 2018 to August 2019. Check mark = fragments were sampled from the colony; NA = not sampled; PM = partial mortality; PB = partial bleaching; R = recovery; M = mortality. Mortality and bleaching percentages are reported relative to initial colony condition; recovery percentages are reported relative to previous sampling time point.

| Colony ID | Summer  2018 | Fall  2018 | Winter  2019 | Spring  2019 | Summer  2019 |
| --- | --- | --- | --- | --- | --- |
| *Pocillopora acuta* |  |  |  |  |  |
| PA1 | ✓ | ✓ PM (~90%) | M / NA | NA | NA |
| PA3 | ✓ | ✓ | ✓ | ✓ | NA |
| PA5 | ✓ | NA | NA | NA | NA |
| PA7 | ✓ | ✓ | ✓ | ✓ | ✓ |
| PA9 | ✓ | ✓ PM (~75%) | ✓ PM (~90%) | M / NA | NA |
| PA10 | ✓ | ✓ PM (~50%) | ✓ PM (~80%) | ✓ PM (~85%) | ✓ R (Spring + ~5%) |
| PA11 | NA | ✓ PB (~5%) | ✓ PM (~20%) | ✓ PM (~20%) | ✓ R (Spring + ~15%) |
| PA13 | NA | ✓ | ✓ | ✓ PM (~30%) | ✓ PM (~30%) |
| PA15 | NA | NA | ✓ PM (~20%) | ✓ PM (~30%) | ✓ PM (~80%) |
| PA16 | NA | NA | ✓ | ✓ PM (~10%) | ✓ PM (~10%) |
| *Acropora nana* |  |  |  |  |  |
| AN1 | ✓ | ✓ | ✓ PM (50%) | ✓ PM (75%) | ✓ PM (75%) |
| AN3 | ✓ | ✓ | ✓ | ✓ | ✓ |
| AN5 | ✓ | ✓ | ✓ | ✓ | NA |
| AN6 | ✓ | ✓ | ✓ | ✓ | ✓ |
| AN7 | ✓ | ✓ | ✓ PM (80%) | ✓ PM (80%) | NA |
| AN8 | ✓ | ✓ | ✓ | ✓ | ✓ |
| AN11 | NA | NA | NA | NA | ✓ |
| AN14 | NA | NA | NA | NA | ✓ |
| *Porites lutea* |  |  |  |  |  |
| PL1 | ✓ | ✓ | ✓ | ✓ | ✓ |
| PL2 | ✓ | ✓ | ✓ | ✓ | ✓ |
| PL3 | ✓ | ✓ | ✓ | ✓ | ✓ |
| PL4 | ✓ | ✓ | ✓ | ✓ | ✓ |
| PL6 | ✓ PM (~5%) | ✓ PM (~5%) | ✓ PM (~5%) | ✓ PM (~5%) | ✓ PM (~5%) |
| PL10 | ✓ | ✓ | ✓ | ✓ | ✓ |

**Table S5.** Nutrient data (mean ± SD) from a warmed and thermally variable reef (Outlet reef) and a thermally stable reef (Wanlitung reef) in southern Taiwan, from May 2018 to June 2019. Parameters measured included 5-day biological oxygen demand (BOD_5_), nitrate (NO_3_^-^), nitrite (NO_2_^-^), phosphate (PO_4_^3-^) and ammonia (NH_3_); all data are shown in mg/L.

| Sampling date | BOD_5_ | | NO_3_^-^ | | NO_2_ | | PO_4_^3-^ | | NH_3_ | |
| --- | --- | --- | --- | --- | --- | --- | --- | --- | --- | --- |
|  | Outlet | Wanlitung | Outlet | Wanlitung | Outlet | Wanlitung | Outlet | Wanlitung | Outlet | Wanlitung |
| May 25, 2018 | 0.6 | 0.6 | 0.002 | 0.002 | 0.006 | 0.008 | 0.003 | 0.002 | 0.01 | 0.015 |
| July 27, 2018 | 1.0 | 1.3 | 0.017 | 0.029 | 0.001 | 0.001 | 0.002 | 0.002 | 0.015 | 0.016 |
| September 5, 2018 | 0.6 | 0.7 | 0.048 | 0.029 | 0.001 | 0.001 | 0.007 | 0.007 | 0.027 | 0.032 |
| October 21, 2018 | 1.5 | 1.2 | <0.002 | <0.002 | 0.052 | 0.017 | 0.011 | 0.006 | 0.015 | 0.018 |
| November 16, 2018 | 0.6 | 0.6 | 0.012 | 0.011 | 0.001 | 0.001 | 0.011 | 0.003 | 0.031 | 0.029 |
| December 20, 2018 | 0.7 | 0.7 | 0.019 | 0.014 | <0.001 | <0.001 | 0.003 | 0.003 | 0.012 | 0.013 |
| January 24, 2019 | 1.5 | 1.6 | 0.023 | 0.016 | 0.001 | 0.001 | 0.017 | 0.095 | 0.019 | 0.013 |
| March. 18, 2019 | 1.5 | 1.2 | 0.019 | 0.019 | 0.001 | 0.001 | 0.003 | <0.002 | 0.012 | 0.015 |
| April 22, 2019 | 1.2 | 0.8 | <0.003 | <0.003 | <0.001 | <0.001 | <0.002 | <0.002 | 0.022 | 0.024 |
| May 23, 2019 | 0.8 | 0.8 | 0.022 | 0.021 | 0.001 | 0.001 | 0.005 | 0.003 | 0.018 | 0.015 |
| June 28, 2019 | 1.2 | 2.6 | 0.021 | 0.016 | 0.001 | 0.001 | 0.003 | <0.003 | 0.01 | 0.01 |
| Mean | **1.0** | **1.1** | **0.017** | **0.014** | **0.006** | **0.003** | **0.006** | **0.011** | **0.017** | **0.018** |
| Standard deviation | **0.4** | **0.6** | **0.014** | **0.010** | **0.015** | **0.005** | **0.005** | **0.028** | **0.007** | **0.007** |
